# Near-Death Experience Memories Include More Episodic Components Than Flashbulb Memories

**DOI:** 10.3389/fpsyg.2020.00888

**Published:** 2020-05-13

**Authors:** Helena Cassol, Estelle A. C. Bonin, Christine Bastin, Ninon Puttaert, Vanessa Charland-Verville, Steven Laureys, Charlotte Martial

**Affiliations:** ^1^Coma Science Group, GIGA-Consciousness, University of Liège, Liège, Belgium; ^2^Centre du Cerveau^2^ (Centre Intégré Pluridisciplinaire de l’Étude du Cerveau, de la Cognition et de la Conscience), University of Liège, Liège, Belgium; ^3^GIGA-CRC In vivo Imaging, University of Liège, Liège, Belgium

**Keywords:** autobiographical memory, near-death experience, flashbulb memory, autobiographical interview, memory ߝ consolidation

## Abstract

Memories of near-death experiences (NDEs) are recalled as “realer” than memories of other real or imagined events. Given their rich phenomenology, emotionality and consequentiality, it was hypothesized that they could meet some aspects of the definition of flashbulb memories. We aimed to identify and compare the episodic and non-episodic information provided in verbal recollections of NDE, flashbulb, and control autobiographical memories. The phenomenological characteristics and centrality of the memories were also compared. Twenty-five participants who had lived a NDE in a life-threatening situation were interviewed and completed the Memory Characteristics Questionnaires as well as the Centrality of Event Scale for their NDE, a flashbulb and another autobiographical memory used as control. Overall, transcribed NDE verbal recollections included a higher overall amount of details and more internal/episodic information than control autobiographical and flashbulb memories. Moreover, flashbulb memories were associated to a lower intensity of feelings while remembering and a lower personal importance, and are less reactivated and less susceptible to be remembered from a first person perspective compared to NDE and control autobiographical memories. Finally, NDE memories are the most central memories to experiencers’ identity, followed by control autobiographical and then by flashbulb memories. These findings corroborate previous studies highlighting the impact and uniqueness of NDE memories.

## Introduction

Near-death experiences (NDEs) have been defined as psychological events usually occurring when people are close to death or in situations of intense danger (Greyson, 2000b). These subjective experiences contain some typical and recurrent features such as the vision of a bright light, out-of-body experiences (OBEs) or coming to a border or a point of no return (Greyson, 1983; Martial et al., 2017a; Cassol et al., 2018). Long regarded as taboo and out of reach for science, the scientific study of the phenomenon emerged only four decades ago but recently led to an increasing number of empirical research (e.g., Van Lommel et al., 2001; Palmieri et al., 2014; Martial et al., 2018, 2019a,b). NDEs are of particular clinical relevance given their frequency of appearance (i.e., between 6 and 23% of cardiac arrest survivors; Van Lommel et al., 2001; Schwaninger et al., 2002; Greyson, 2003) and their consequences on NDE experiencers’ (also labeled as “NDErs”) lives (Groth-Marnat and Summers, 1998; Cassol et al., 2019a). Numerous aftereffects have been reported, such as the development of a higher spirituality, less materialistic values or a reduced fear of death (Noyes, 1980; Groth-Marnat and Summers, 1998; Knoblauch et al., 2001).

Previous studies have highlighted the uniqueness of NDE memories within autobiographical memory (i.e., memories about an individual’s life; Williams et al., 2008). NDE memories were found to contain more details (e.g., sensory, emotional, and self-referential) than memories of other real and imagined events, and memories of a period of coma or impaired consciousness following an acquired severe brain dysfunction without NDE (Thonnard et al., 2013). Thus, it was suggested that they could not be considered as imagined event memories. Other studies have found that richer NDEs (i.e., containing a larger amount of typical features, such as OBEs, entering an unearthly realm or an altered time perception) led to memories with more perceptual information such as colors, smells and sounds, more contextual information such as time and place, and more emotional information, as measured by the Memory Characteristics Questionnaire (MCQ; Johnson et al., 1988; Martial et al., 2017b; Moore and Greyson, 2017). At first sight, the important amount of phenomenological details contained in their recollections might seem counter-intuitive given that NDEs are assumed to occur during an altered state of consciousness and are classically associated with a critical or confused state (e.g., Van Lommel et al., 2001). In an effort to explain the special nature of NDE memories, different theories have been put forth. It was suggested that the significant emotionality associated with these mental events could enhance the quantity of sensory details stored in memory (Schaefer and Philippot, 2005). Besides, emotional events are more likely to be frequently rehearsed, therefore potentially increasing the amount of memorized characteristics (Thonnard et al., 2013).

Given their emotionality and consequentiality (i.e., the consequences of the event or aftermath; e.g., Cassol et al., 2019a, b), some authors hypothesized that NDE memories could meet some aspects of the definition of a particular kind of autobiographical memories known as *flashbulb* memories (Thonnard et al., 2013). Flashbulb memories refer to very vivid and long-lasting memories of the circumstances in which one learned about a shocking public event (Brown and Kulik, 1977). Studies on flashbulb memories have been focusing on landmark events such as the September 11 terrorist attacks or the death/assassination of famous people (Finkenauer et al., 1998; Curci et al., 2001; Tinti et al., 2009). A prominent feature of these memories is that they display high ratings of belief in accuracy/subjective certainty (Brown and Kulik, 1977; Kraha and Boals, 2014) and that people can usually remember some of their personal aspects for several decades (Berntsen and Thomsen, 2005), for example the place they were when they heard the news, who they were with, the clothes they were wearing, or the emotions felt at the time of the event (Luminet and Curci, 2009). Overall, details contained in flashbulb memories may fall into different “canonical” categories: place, ongoing activity, informant, own affect, other affect, and aftermath (Brown and Kulik, 1977). The level of consequentiality associated with flashbulb memories and the underlying memory processes, however, remain debated. Pioneers in the area have suggested that these memories do not imply the existence of a special memory mechanism and include the notion of consequentiality as a key element in the formation of these memories, along with novelty, surprise, emotionality and overt rehearsal [i.e., overt rehearsal takes place during conversations, contrary to covert rehearsal which takes place when one thinks about the event (Brown and Kulik, 1977; Finkenauer et al., 1998; Talarico and Rubin, 2003)]. However, results from other studies suggest different underlying memory processes for memories of learning about public news (such as flashbulb memories) and memories for private events (i.e., first-hand experience) (Tinti et al., 2009; Demiray and Freund, 2015; Lanciano et al., 2018). For example, first-hand experiences would show a higher consequentiality and life impact, and would have a protective function because their content is related to the individual’s personal identity and well-being (Pillemer, 2009).

Generally speaking, narratives of autobiographical memories are known to be multifaceted in content and to include both episodic (i.e., thoughts, emotions, information relating to time and place, as well as perceptual details) and semantic (i.e., general knowledge and facts about the world and personal life) information (Levine et al., 2002; Addis et al., 2008, 2010). Given that episodic and semantic memory are thought to rely on different neural processes (Moscovitch et al., 2005), Levine et al. (2002) have developed an Autobiographical Memory Interview (AI) which includes a reliable coding scheme enabling the distinction between episodic and non-episodic details comprised in verbalized autobiographical memories. In their AI coding scheme, *episodic details* (scored by using categories adapted from the MCQ) refer to the central event and its emotional, perceptual, temporal and spatial characteristics, and *non-episodic details* relate to repetitions, metacognitive statements, and information that does not pertain to the main event or that is not contextually bounded, such as general knowledge or facts. While the former seem essential for high-fidelity representation of personally experienced events, the latter rather contribute to the coherence and continuity of self-knowledge and identity through time (Levine et al., 2002). To date, the AI scoring scheme has been used in a wide range of studies related to memory processes in aging (e.g., Levine et al., 2002), psychiatric conditions (e.g., Moscovitch et al., 2018), neurodegeneration (e.g., Bastin et al., 2013) or lesion cases (e.g., Steinvorth et al., 2005). However, no study has yet investigated the episodic and semantic composition of NDE verbal recollections as well as the comparison with those of flashbulb memories. Therefore, the primary aim of this study was to (1) assess whether the episodic and non-episodic information comprised in verbal recollections of NDE, flashbulb and control autobiographical memories differ and (2) further compare their phenomenological characteristics using the sMCQ (a short version of the MCQ; Johnson et al., 1988; D’Argembeau and Van der Linden, 2008) and their consequentiality by means of the Centrality of Event Scale (CES; Berntsen and Rubin, 2006).

## Materials and Methods

### Participants

NDErs were recruited via the websites, appearances in local media and publications of the International Association for Near-Death Studies (IANDS France), the Coma Science Group (University of Liège, Belgium). Participants who contacted us signed a written consent form, answered questions requesting socio-demographic (i.e., gender and age at interview) as well as clinical information (i.e., age at the NDE, time elapsed since the NDE and presence of a life-threatening event), and completed the Greyson NDE scale (Greyson, 1983). This 16-item multiple-choice validated scale enables a standardized identification of a NDE (Greyson, 1983; Lange et al., 2004) as well as the quantification of its intensity (i.e., total score ranging from 0 to 32). For each of the 16 items, the scores are arranged on an ordinal scale ranging from 0 to 2 (i.e., 0 = *not present*, 1 = *mildly or ambiguously present*, and 2 = *definitively present*).

Ultimately, we included 25 participants whose experience had been lived in a life-threatening situation and who met the accepted criteria of a NDE (i.e., Greyson NDE scale’s total score ≥ 7). The study was approved by the Ethics Committee of the Faculty of Medicine of the University of Liège.

### Experimental Task

#### Screening Tasks

The first step of the face-to-face interview consisted of a screening assessment in order to detect and exclude memory biases related to negative mood as well as mild cognitive impairments.

Regarding negative mood, the 25 NDErs completed the Positive and Negative Affect Schedule (PANAS; Watson et al., 1988). The PANAS consists of 20 emotion words, with 10 loading on the Positive Affect factor and 10 on the Negative Affect one. This scale assesses participants’ baseline mood levels. The positive subscale (PANAS+) includes words such as “alert,” “inspired,” or “enthusiastic,” whereas the negative subscale (PANAS−) includes words such as “distressed,” “upset,” or “guilty.” Participants rate the degree to which they endorse each item on a rating scale ranging from 1 = *very slightly or not at all to* 5 = *extremely*. Items are then summed to create a score for each factor. Higher scores represent greater endorsement of the construct.

Potential mild cognitive impairments were assessed using the Montreal Cognitive Assessment (MoCA; Nasreddine et al., 2005). This scale includes 30 items assessing multiple cognitive domains: *short-term memory* (5 points); *visuospatial abilities* via clock drawing (3 points) and a cube copy task (1 point); *executive functioning* via an adaptation of Trail Making Test Part B (1 point), phonemic fluency (1 point) and verbal abstraction (2 points); *attention, concentration and working memory* via target detection (1 point), serial subtraction (3 points), digits forward (1 point) and digits backward (1 point); *language* via confrontation naming with low-familiarity animals (3 points) and repetition of complex sentences (2 points); and *orientation to time and place* (6 points). Items are summed to create a score and the clinical cut-off score is equal to 26.

#### Autobiographical Memory Interview (Levine et al., 2002)

Once participants were screened for mood and for memory impairments, the interviews were carried out based on the probing and scoring recommendations of the Autobiographical Memory Interview (AI) by Levine et al. (2002). We conducted a semi-structured interview in a quiet room for each participant. Specifically, participants were asked to freely describe in considerable detail three target memories dating (without restriction regarding the recall time), as far as possible, to the same period: (1) the memory of their NDE, (2) a flashbulb memory (they were provided with a definition and examples; i.e., memories of the circumstances in which one first learned of a very surprising and consequential or emotionally arousing event such as September 11 terrorist attacks), and (3) an autobiographical memory. Each event had to be temporally and contextually specific, that is, occurring over minutes or hours but not exceeding a day. The order in which the memories were to be recalled was randomized. According to the AI guidelines, two further levels of recall may be needed in case of a lack of key information: (1) general and (2) specific probing. General probes are non-specific cues aiming to encourage full description (e.g., “Can you tell me more about it?”). Specific probes cue precise aspects of the event in order to determine if participants remember more information (e.g., “Where did this event take place?”). Nevertheless, no specific probe was employed in the present study due to substantial recalls by our participants. Narratives were audio-recorded, transcribed and analyzed using the established manual scoring procedure, allowing to separate episodic details (i.e., description of the event, sensory or mental state details specific to the event; “internal details”) from non-episodic details (i.e., semantic or factual statements, or other details not specific to the event; “external details”) (Levine et al., 2002).

#### Questionnaires

Finally, after each memory recall, participants completed a short version of the Memory Characteristics Questionnaire (sMCQ; D’Argembeau and Van der Linden, 2008; adapted from Johnson et al., 1988) and the Centrality of Event Scale (CES; Berntsen and Rubin, 2006). The former assesses memory clarity, sensory details, self-referential and emotional information, reactivation frequency, and confidence in the memory by means of 16 items (each rated on a 1–7 point Likert scale). The latter is a 20-item instrument that measures how central an event is to a person’s identity and life story. All items are rated on a 5-point scale ranging from 1 = *totally disagree* to 5 = *totally agree*. The three groups of items provide an assessment of whether an event has become a reference point for the generation of expectations to other events in the respondent’s life story, whether an event is considered as a central component of the person’s identity, and whether the event is considered as a turning point in one’s life story, respectively.

### Analysis

Data analyses were carried out using Jamovi statistical software (1.1.9.0).

#### Study Sample

Greyson NDE scale total scores, age at NDE and age at interview are summarized as mean ± standard deviation (SD). Distributions being skewed, results at the PANAS and the MoCA are summarized as median (inter-quartile range; IQR), and differences for time since event (NDE vs. flashbulb vs. autobiographical) were analyzed using Friedman tests and expressed as median (IQR) and average rank.

#### Autobiographical Memory Interview

Transcribed recalls were segmented into “details,” or “segments.” A detail is defined as “a unique occurrence, observation, or thought, generally expressed as a grammatical clause” (Levine et al., 2002). A segment can be defined as a sentence or part of a sentence that conveys information. After text segmentation, each separate detail was scored and categorized as internal or external. Internal details pertain to the main event and are episodic in nature, whereas external details are not directly related to the main event and may correspond to semantic information. Internal details are subdivided into: (1) event details, which describe the unfolding of the story (e.g., happenings, persons involved, reactions/emotions of oneself or other people, one’s clothing, the weather); (2) the time (e.g., life epoch, day, month, year, season, and clock time); (3) the place (i.e., any information that involves localization in space, such as a city, a street, a building, a room); (4) perceptual details (i.e., visual, olfactory, tactile, gustatory, auditory information, proprioceptive, or nociceptive information); and (5) thoughts and emotions (relating to the mental state of the subject). Apart from semantic information, repetitions of information and other unrelated details (i.e., time, place, event, perceptual and emotional details that do not directly pertain to the main event) are classified as external. Two independent raters (HC and EB) analyzed each transcribed recall by scoring each detail. Scoring reliability was assessed using intra-class correlation (ICC) coefficients, showing that both raters scored the narratives in a highly reliable manner (ICC_internal details_ = 0.99; ICC_external details_ = 0.98). Residual discrepancies between raters were discussed later on to reach a final classification for all details. Based on this final classification, all subtypes of details generated spontaneously were summed across memories.

For each type of memory, the measures of interest were the overall amount of (1) internal and (2) external details. First, we looked at potential associations between the number of reported details and demographic variables (i.e., age at NDE, age at event and time since event) using Pearson’s correlations, as well as between these details and the MoCA total scores using Spearman’s correlations given that they were not normally distributed. Second, we looked at the difference in the amount of internal and external details reported for each type of memory. Variables being normally distributed, differences between groups were assessed using two-ways repeated measures analysis of variance (rmANOVAs), with the type of details (interval vs. external) and the type of memory (NDE vs. flashbulb vs. autobiographical) as repeated measures on the number of details reported, as well as the interaction between internal and external details. In case of violation of sphericity, we used a Greenhouse–Geisser correction. Results were considered significant at *p* = 0.05. In case of significant results, we performed *post hoc* comparisons using Tukey HSD, setting the level of significance at 0.017 after Bonferroni correction. Regarding additional measures, we looked at the effect of the memory type on the amount of subtypes of internal and external memory details (i.e., event, perceptual, emotional and semantic details, as well as time, place and repetitions). Distributions being skewed, differences between groups were assessed using Friedman tests and results were considered significant at *p* = 0.004 after Bonferroni correction. In case of significant results, we performed *post hoc* comparisons using Wilcoxon signed-rank tests, setting the level of significance at 0.017 after Bonferroni correction.

#### Questionnaires

CES total scores were analyzed using an rmANOVA. Answers at the sMCQ being ordinal data measures, memories (NDE vs. flashbulb vs. autobiographical) were compared using a Friedman test, setting the level of significance at 0.003 after Bonferroni correction. In case of significant results, we performed *post hoc* comparisons using Tukey HSD tests for rmANOVAs and Wilcoxon signed-rank tests for Friedman tests, setting the level of significance at 0.017 after Bonferroni correction. Additionally, we examined associative strength between CES total scores and reported details (i.e., internal and external score) as well as sMCQ items using Spearman’s correlations.

## Results

### Study Sample

Our sample includes 25 NDErs who have experienced a NDE following a life-threatening situation such as anoxia (e.g., cardiac arrest, near-drowning; *n* = 8), trauma (e.g., motor vehicle accident, falls; *n* = 6) or other events (non-traumatic events such as complication during surgery; *n* = 11). Participants’ negative mood was not higher than normal (i.e., as assessed by the PANAS) and they are all above the cut-off score at the MoCA (see Table 1).

**TABLE 1:** Demographic and clinical information for NDErs (*n* = 25; 10 females).

**Age at interview (in years)**	
Mean ± SD	59 ± 12
**Age at NDE (in years)**	
Mean ± SD	31 ± 20
**Reported time since NDE (in years)**	
Mean ± SD	28 ± 19
**Greyson NDE scale total score**	
Mean ± SD	15 ± 5
**PANAS +**	
Median (IQR)	35 (29–39)
**PANAS −**	
Median (IQR)	12 (10–16)
**MoCA**	
Median (IQR)	29 (28–30)

*NDE, near-death experiences; NDErs, near-death experiencers; SD, standard deviation; IQR, inter-quartile range.*

All participants were able to recall a flashbulb memory and an autobiographical memory that happened around the same time as the NDE. Recalled memories did not differ regarding the number of years elapsed since the event (median_NDE_ = 27, IQR = 10–45, average rank = 2.24; median_Flashbulb_ = 17, IQR = 15–27, average rank = 1.68; median_Autobiographical_ = 26, IQR = 12–42, average rank = 2.08; [*X*^2^(2) = 4.622, *p* = 0.099, kendall coefficient = 0.092]). Recalled flashbulb memories were the following: the assassination of J. F. Kennedy (*n* = 1), the first steps on the moon (*n* = 5), the fall of the Berlin wall (*n* = 2), death of King Baudouin of Belgium (*n* = 3), death of Princess Diana (*n* = 1), September 11 terrorist attacks (*n* = 8), Liège shooting attack (*n* = 4), and Paris terrorist attacks (*n* = 1). Demographic and clinical information of the entire sample can be found in Table 1.

#### Autobiographical Memory Interview

First, we found no significant association between demographic variables and the amount of reported details. Precisely, the age at interview was not correlated with the amount of internal details comprised in NDE (*r* = −0.188, *p* = 0.367), flashbulb (*r* = −0.063, *p* = 0.764) and autobiographical (*r* = 0.175, *p* = 0.402) memories, as well as the amount of external details of NDE (*r* = 0.051, *p* = 0.808) flashbulb (*r* = −0.230, *p* = 0.270) and autobiographical memories (*r* = 0.055, *p* = 0.794). The age at event was not correlated with the amount of internal details comprised in NDE (*r* = −0.292, *p* = 0.156), flashbulb (*r* = 0.243, *p* = 0.242) and autobiographical memories (*r* = −0.077, *p* = 0.715), as well as the amount of external details of NDE (*r* = 0.051, *p* = 0.808) flashbulb (*r* = −0.126, *p* = 0.548) and autobiographical memories (*r* = 0.024, *p* = 0.910). Finally, the time since event was not correlated with the amount of internal details comprised in NDE (*r* = 0.174, *p* = 0.405), flashbulb (*r* = −0.297, *p* = 0.149) and autobiographical memories (*r* = 0.199, *p* = 0.340), as well as the amount of external details of NDE (*r* = 0.006, *p* = 0.977), flashbulb (*r* = −0.037, *p* = 0.861) and autobiographical memories (*r* = −0.283, *p* = 0.171).

Second, we found no significant association between the MoCA total scores and the amount of internal details comprised in NDE (rho = −0.334, *p* = 0.103), flashbulb (*r* = −0.276, *p* = 0.183), and autobiographical (rho = 0.061, *p* = 0.771), memories, as well as the amount of external details of NDE (*r* = −0.378, *p* = 0.062) flashbulb (*r* = 0.369, *p* = 0.188), and autobiographical memories (*r* = −0.199, *p* = 0.341).

Finally, the rmANOVA revealed a main effect of the type of detail [*F*(1,24) = 32.4, *p* < 0.001, $\eta_{\text{p}}^{2}$ = 0.575], NDErs recalled a higher overall amount of internal details (*M* = 29.1, *SD* = 26) compared to external ones (*M* = 13.4, *SD* = 11.8). We also found a main effect of the type of memory [*F*(1.22,29.35) = 28.2, *p* > 0.001, $\eta_{\text{p}}^{2}$ = 0.54]. The overall amount of details was higher for the NDE (*M* = 36.4, *SD* = 30.2) compared to the flashbulb (*M* = 12.5, *SD* = 8.47; *t* = 6.813, *p* = 0.001) and the autobiographical memory (*M* = 14.8, *SD* = 8.74; *t* = −6.151, *p* < 0.001), which did not differ from each other (*t* = 0.662, *p* = 0.786). Lastly, the interaction between the type of detail and the type of memory was also significant [*F*(1.42,34.10) = 19.3, *p* < 0.001, $\eta_{\text{p}}^{2}$ = 0.445]. Specifically, the number of internal details reported for NDE memories (*M* = 53, *SD* = 32.7) is higher than the internal details reported for flashbulb (*M* = 16, *SD* = 8.90; *t* = 8.660, *p* < 0.001) and autobiographical memories (*M* = 18.2, *SD* = 5.29; *t* = −8.127, *p* < 0.001), and higher than the amount of external details reported for NDE (*M* = 19.8, *SD* = 14.6; *t* = 8.420, *p* < 0.001), flashbulb (*M* = 9.08, *SD* = 6.53; *t* = 9.402, *p* < 0.001) and autobiographical memories (*M* = 11.4, *SD* = 10.02; *t* = 8.897, *p* < 0.001). The other conditions did not significantly differ (all *p*s > 0.14).

Differences in episodic and non-episodic subcomponents of NDE, flashbulb and autobiographical memories can be found in Table 2. Results indicated a main effect of the memory type on the amount of event and perceptual internal details (see Table 2). Specifically, *post hoc* comparisons indicated that NDE memories contained significantly more internal event details than autobiographical (*V* = 12, *p* < 0.001) and flashbulb memories (*V* = 19, *p* < 0.001). Autobiographical and flashbulb memories did not differ (*V* = 80, *p* = 0.223). NDE memories also showed higher ratings for internal perceptual details as compared to autobiographical (*V* = 3.5, *p* < 0.001) and flashbulb memories (*V* = 246, *p* < 0.001). Once again, autobiographical and flashbulb memories did not differ (*V* = 54.5, *p* = 0.775). No other significant difference was observed. Although not significant when considering Bonferroni correction for multiple comparisons, there was a trend for higher emotional internal details associated with the NDE compared to other types of memories.

**TABLE 2:** Episodic/internal and non-episodic/external subcomponents of NDE (*n* = 25), flashbulb (*n* = 25), and autobiographical memories (*n* = 25).

**AI details**	**NDE median (IQR)**	**NDE average rank**	**Flashbulb median (IQR)**	**Flashbulb average rank**	**Autobio median (IQR)**	**Autobio average rank**	***X*^2^**	**Df**	***p*-value**	**Kendall coefficient**
**Internal**										
Event	20 (9–33)	2.62	6 (3–10)	1.50	10 (4–12)	1.88	18.431	2	< 0.004*	0.369
Time	2 (1–3)	2.10	1 (1–2)	1.64	2 (1–3)	2.26	6.241	2	0.044	0.125
Place	1 (0–2)	2.02	1 (1–2)	2.18	1 (0–2)	1.80	2.844	2	0.241	0.057
Perceptual	7 (3–12)	2.76	0 (0–2)	1.58	0 (0–2)	1.66	27.175	2	< 0.004*	0.544
Emotional	10 (5–16)	2.48	3 (2–6)	1.70	6 (2–8)	1.82	9.800	2	0.007	0.196
**External**										
Event	7 (1–13)	2.36	2 (1–3)	1.62	3 (1–6)	2.02	7.708	2	0.021	0.154
Time	0 (0–0)	2.06	0 (0–0)	2.00	0 (0–0)	1.94	0.750	2	0.687	0.015
Place	0 (0–0)	1.98	0 (0–0)	1.96	0 (0–0)	2.06	0.378	2	0.828	0.057
Perceptual	0 (0–1)	2.16	0 (0–0)	1.80	0 (0–0)	2.04	7.000	2	0.030	0.140
Emotional	1 (0–5)	2.16	1 (0–2)	1.96	0 (0–2)	1.88	1.489	2	0.476	0.030
Semantic	1 (0–3)	1.98	3 (1–4)	2.24	1 (0–2)	1.78	3.325	2	0.190	0.067
Repetition	3 (1–4)	2.32	2 (1–2)	1.88	1 (0–3)	1.80	4.722	2	0.094	0.094

Results for the AI (autobiographical memory interview) details are considered statistically significant at p < 0.004 after Bonferroni correction. *Results are significant. NDE, near-death experience; IQR, inter-quartile range; Df, degree of freedom.

### Questionnaires

#### Short Memory Characteristics Questionnaire

Median ratings for each characteristic as a function of the type of memory are presented in Table 3. Results indicated a between-memory difference in the (1) feeling of mentally reliving the event, (2) sensation of feeling the emotions felt during the event while remembering, (3) visual perspective taken while remembering, (4) emotions felt at the time of the event (i.e., valence), (5) personal importance attached to the event, and (6) reactivation frequency. Memories did not differ in the amount of sensory details, clarity (i.e., time, location and coherence), confidence in the memory as well as remembering one’s own actions/words/thoughts. *Post hoc* comparisons indicated that the feeling of mentally reliving the event is higher in autobiographical memories as compared to flashbulb memories (*V* = 187, *p* = 0.002). NDE memories did not differ from autobiographical (*V* = 32, *p* = 0.361) and flashbulb memories (*V* = 144, *p* = 0.049). The sensation of feeling the emotions felt during the event while remembering is higher in NDE and autobiographical memories compared to flashbulb memories (*V* = 192, *p* = 0.001 and *V* = 20, *p* < 0.001, respectively), but did not differ between NDE and autobiographical memories (*V* = 37.5, *p* = 0.109). Regarding visual perspective, flashbulb memories showed lower scores than NDE (*V* = 162.5, *p* < 0.001) and autobiographical memories (*V* = 4.5, *p* < 0.001) indicating that they were less susceptible to be remembered from a first-person perspective. Difference between NDE and autobiographical memories did not reach significance (*V* = 24, *p* = 0.438). Flashbulb memories also showed lower scores for the valence as compared to NDE (*V* = 207.5, *p* < 0.001) and autobiographical memories (*V* = 232, *p* = 0.001; NDE and autobiographical memories did not differ, *V* = 74.5, *p* = 0.171), indicating that the latter were more positive in average. NDE and autobiographical memories also scored higher than flashbulb memories in terms of personal importance (*V* = 250, *p* < 0.001 and *V* = 7.5, *p* < 0.001, respectively). On the other hand, difference between NDE and autobiographical memories did not reach significance (*V* = 30.5, *p* = 0.307). Finally, NDE and autobiographical memories were more frequently shared and reactivated than flashbulb memories (*V* = 211.5, *p* < 0.001 and *V* = 194, *p* < 0.001, respectively). Once again, difference between NDE and autobiographical memories did not reach significance (*V* = 73.5, *p* = 0.793).

**TABLE 3:** Score differences between NDE (*n* = 25), flashbulb (*n* = 25), and autobiographical (*n* = 25) memories on the sMCQ.

**sMCQ Items**	**NDE median (IQR)**	**Average rank**	**Flashbulb median (IQR)**	**Average rank**	**Autobio median (IQR)**	**Average rank**	***X*^2^**	**Df**	***p*-value**	**Kendall coefficient**
Reexperiencing	6 (6–7)	2.20	5 (3–6)	1.50	7 (6–7)	2.30	12.500	2	0.002*	0.250
Visual details	7 (6–7)	2.20	6 (6–7)	1.76	7 (6–7)	2.04	4.863	2	0.088	0.097
Other sensory details	6 (5–7)	2.36	4 (2–5)	1.60	6 (4–7)	2.04	9.455	2	0.009	0.189
Location	7 (7–7)	2.10	7 (7–7)	2.02	7 (6–7)	1.88	1.676	2	0.433	0.034
Time	4 (1–6)	1.86	6 (5–6)	2.32	5 (2–6)	1.82	4.437	2	0.109	0.089
Coherence	7 (7–7)	2.22	7 (5–7)	1.72	7 (6–7)	2.06	7.244	2	0.027	0.145
Verbal component	5 (4–7)	2.12	5 (4–6)	1.92	6 (3–6)	1.96	0.903	2	0.637	0.018
Feeling emotions	6 (5–7)	2.12	5 (3–6)	1.48	7 (6–7)	2.40	13.728	2	0.001*	0.275
Real/imagined	7 (7–7)	2.08	7 (7–7)	1.96	7 (7–7)	1.96	1.200	2	0.548	0.024
One’s own actions	7 (6–7)	2.28	6 (4–7)	1.58	7 (6–7)	2.14	11.064	2	0.004	0.221
One’s own words	7 (5–7)	2.18	4 (3–7)	1.80	6 (4–7)	2.02	2.563	2	0.278	0.051
One’s own thoughts	7 (7–7)	2.38	6 (4–7)	1.74	6 (6–7)	1.88	8.448	2	0.015	0.169
Visual perspective	7 (7–7)	2.28	5 (1–6)	1.30	7 (7–7)	2.42	27.382	2	< 0.003*	0.548
Valence	7 (6–7)	2.24	3 (2–4)	1.24	7 (6–7)	2.52	28.658	2	< 0.003*	0.573
Personal importance	7 (6–7)	2.42	3 (2–4)	1.30	7 (5–7)	2.28	22.707	2	< 0.003*	0.454
Reactivation frequency	6 (5–6)	2.38	3 (2–4)	1.34	5 (5–6)	2.28	20.835	2	< 0.003*	0.416

IQR, inter-quartile range; Df, degrees of freedom. *Results are significant. Results are considered statistically significant at p < 0.003 after Bonferroni correction.

#### Centrality of Event Scale

There was a significant effect of the memory type on the reported centrality [*F*(2,48) = 48.256, *p* < 0.001, $\eta_{\text{p}}^{2}$ = 0.668]. *Post hoc* comparisons indicated that the mean score for the NDE memory (*M* = 81.4, *SD* = 15.767) was significantly higher than for the autobiographical memory (*M* = 62, *SD* = 23.429) [*F*(1,24) = 12.767, *p* = 0.002] as well as for the flashbulb memory (*M* = 35.36, *SD* = 12.783) [*F*(1,24) = 115.525, *p* < 0.001]. Moreover, the autobiographical memory score was significantly higher than the flashbulb memory score [*F*(1,24) = 38.149, *p* < 0.001]. Additionally, a significant positive correlation was found between CES scores and the number of reported internal details (*r*_s_ = 0.512, *p* < 0.001), but not with the number of external details (*r*_s_ = 0.208, *p* = 0.073).

Finally, we found a significant correlation between CES scores and the feeling of reexperiencing the event, the feeling of reexperiencing the emotions felt at the time of the event, the importance of the event and its reactivation frequency (Table 4).

**TABLE 4:** Association between CES total scores and sMCQ items.

	**Rho**	***S***	***p*-value**
Reexperiencing	0.345	46050	0.002*
Visual details	0.117	62073	0.317
Other sensory details	0.178	57782	0.126
Location	–0.019	71639	0.871
Time	–0.284	90260	0.014
Coherence	0.109	62650	0.353
Verbal component	0.030	68214	0.801
Feeling emotions	0.427	40280	< 0.003*
Real/imagined	0.196	56539	0.092
One’s own actions	0.205	55898	0.078
One’s own words	0.162	58881	0.164
One’s own thoughts	0.246	53000	0.033
Visual perspective	0.322	47666	0.005
Valence	0.281	50543	0.015
Personal importance	0.713	20145	< 0.003*
Reactivation frequency	0.399	42191	< 0.003*

*Results are significant. Results are considered statistically significant at p < 0.003 after Bonferroni correction.

## Discussion

Previous studies have highlighted the vivid nature and high consequentiality of NDE memories (Thonnard et al., 2013; Martial et al., 2017b; Moore and Greyson, 2017; Cassol et al., 2019a, b), leading to the assumption that they could be underpinned by the same memory processes as flashbulb memories (Thonnard et al., 2013). Therefore, the aim of this study was to adopt a more fine-grained approach to highlight and compare the episodic and non-episodic contributions to the recall of NDE, flashbulb and control autobiographical events that occurred around the same time. To do so, we used the AI which provides reliable and valid indices of episodic and semantic contributions to personal remote memories.

First, analysis of verbal recollections highlighted that NDE memories include a higher overall amount of details and a higher amount of internal/episodic details than flashbulb and autobiographical memories. This is consistent with previous studies suggesting a particularly high amount of qualitative characteristics associated with NDE memories in comparison with other types of memories (e.g., Thonnard et al., 2013; Moore and Greyson, 2017). Moreover, the difference between the number of internal and external details for NDE memories is significantly larger than the one observed for flashbulb and autobiographical memories. The richness and high amount of overall details (and particularly internal/episodic information) delivered for NDE memories, compared to other more or less notable memories dating from the same period, could be explained by various factors. First, a NDE itself is characterized by an exceptional and unique association between unusual perceptions (such as the impression of leaving the physical body, entering a different dimension or being in an unknown spatio-temporal dimension) and a clear sensorium and intense sensation of “reality” (i.e., comparable to the sense of certainty that accompanies everyday perception; Schwaninger et al., 2002; Dell’Olio, 2010), conditions that are likely to lead to vivid memories. Second, previous studies have highlighted a tendency toward a decline in memory reports (normal forgetting process) in flashbulb memories in comparison with other emotionally arousing events (e.g., Christianson, 1989; Christianson and Engelberg, 1999). Consistency and amplification of memories over time would be conditioned by one’s degree of involvement and the severity of the emotionally arousing event (Neisser et al., 1996; van Giezen et al., 2005). In line with this view, studies including participants who were directly exposed to or personally involved in traumatic events identified different memory patterns than studies addressing flashbulb memories, in which subjects were not personally involved in the emotional event. In fact, some studies have even shown that unlike the emotionally charged traumatic events whose narratives become richer over time, flashbulb memories rather tend to decline (Krinsley et al., 2003; van Giezen et al., 2005). When looking closer at the subtypes of details that have been recalled, we identified more event and perceptual internal details within the NDE memory, as compared to flashbulb and autobiographical memories. Specifically, *event details* describe the unfolding of the story, and *perceptual details* comprise auditory, olfactory, tactile, taste, visual and spatial-temporal (allocentric-egocentric space, body position and duration) information. Some authors suggested that the overall quantity of details comprised in NDE memories could be due to the fact that self-referential information can improve recalling performances (Conway and Dewhurst, 1995). Specifically, self-referential information would enhance the encoding process, the organization in memory, as well as the enrichment of the event by extended knowledge (Conway and Dewhurst, 1995). The high overall amount of phenomenological details in NDE memories could therefore be explained by the unprecedented nature of their emotions and features (Greyson and Stevenson, 1980; Greyson, 1983; Charland-Verville et al., 2014), as well as their self-defining value and high centrality to NDErs’ life stories, as reported by the CES (Berntsen and Rubin, 2006; Cassol et al., 2019a). From a broader cognitive perspective, the high amount of details observed in verbal recollections of NDE memories is interesting as it suggests that people might be able to recall memories of a moment characterized by an altered state of consciousness where the brain and its associated processes are thought to be working with altered capacities. Nevertheless, it is still unclear when exactly these events are experienced as well as when their memory encoding precisely occurs.

Second, we compared the phenomenological characteristics (i.e., sMCQ scores) as well as the consequentiality and centrality (i.e., CES scores) of NDE, flashbulb and autobiographical memories. The analyses revealed that sMCQ scores related to NDEs and autobiographical memories were higher than those of flashbulb memories regarding the sensation of feeling the emotions felt during the event while remembering, their importance and their reactivation frequency. Moreover, the feeling of mentally reliving the event was higher for the autobiographical memory compared to the flashbulb memory. The finding that NDEs are emotionally highly charged and constitute an important part of NDErs’ life story is in line previous reports (e.g., Greyson, 1997; Bianco et al., 2017; Cassol et al., 2019a). On the contrary, the high scores of autobiographical memories might seem unexpected but could find explanation in the fact that some of them were somewhat connected to the NDE. Indeed, NDErs have lived their NDE several decades ago in average and reported difficulties when having to recall another event from the same period. From then on, recalled autobiographical memories were events closely linked to their NDE/coma (i.e., return to their daily lives after a long hospitalization, recovery after their coma) or significant/life-changing events such as a child birth or their encounter with their spouse (i.e., categories of memories that have typically been reported as self-defining in previous studies; e.g., Cassol et al., 2019a). Furthermore, regarding reactivation frequency, it has been shown that shocking memories of personal events are more thought and talked about than news reception memories such as flashbulb events (Pillemer, 2009). Finally, we also noted significant differences regarding the perspective taken during recollection. Autobiographical memories can either be retrieved from the first person perspective, in which the experience is visualized through one’s own eyes, or from the third person perspective, in which the experience is seen through an observer’s eyes (Nigro and Neisser, 1983). While participants tended to see the scene from their own perspective (field perspective) when recalling their NDE and their autobiographical memory, it was less the case for flashbulb memories for which they were more likely to see themselves from an observer’s perspective. Adopted visual perspectives are influenced by different variables and appear to be the consequence of contextual as well as dispositional factors, and/or their interaction (Nigro and Neisser, 1983; Sutin and Robins, 2008). These findings are consistent with previous research (Robinson and Swanson, 1993; Sutin and Robins, 2008) highlighting that first-person memories are generally rated higher on the phenomenological dimensions related to the reliving of a memory. Moreover, it has been shown that the adoption of a third-person perspective could be an avoidance strategy set up to distance the individual from a memory and reduce its emotional intensity (Kenny and Bryant, 2007; Sutin and Robins, 2008). Therefore, the difference in the adopted visual perspective could be due to the emotions felt at the time of the event (i.e., valence), that are overall very positive in NDE as well as in autobiographical memories and more negative in case of flashbulb events. Regarding centrality, answers to the CES revealed that NDE memories had the higher scores, followed by autobiographical memories and then by flashbulb memories. These findings corroborate previous findings and confirm the particular status of NDEs with regard to NDErs’ identities and life stories (Cassol et al., 2019a). The lower scores associated with flashbulb memories highlight the distinction between the consequences that one predicts when hearing about a surprising event and its actual impact on one’s life. These results are consistent with previous research on hearing shocking public events that have shown lower life impact, consequentiality, and personal importance (Pillemer, 2009). In addition, we found that the amount of episodic details provided upon recall, the feeling of reexperiencing the event while remembering, the feeling of reexperiencing the emotions felt at the time of the event and the reactivation frequency of the event in memory were all associated to the reported centrality of the event, regardless of the memory type. It is therefore reasonable to hypothesize that central/landmark events could benefit from a preferential encoding in autobiographical memory and lead to a richer phenomenology.

Finally, we would like to mention limitations to this study. NDErs voluntarily contacted us after calls in the lay media and our sample consequently may suffer from a self-selection bias. Moreover, given the relative scarcity of NDEs and the fact that we had to meet participants personally in accordance to the AI protocol, we were limited in the recruitment of our participants and our sample is relatively small. Therefore, results should be confirmed by larger studies. Besides, human memory is by definition a reconstructive process (Schacter et al., 1998) sensitive to numerous factors such as aging (e.g., Devitt et al., 2017); therefore, the generalizability of our results could also be impacted by the fact that all memories are drawn from a homogenous pool of NDErs. To address this issue, it would be interesting to assess autobiographical and flashbulb memories in healthy controls and to further explore the cognitive processes specific to NDErs, especially since previous studies suggest a specific cognitive profile, including a propensity for dissociation (Greyson, 2000a) and illusory recollections (Martial et al., 2017c). In the present study, we did not control for the production of false memories and the subjective feeling of remembering that may accompany false remembering (i.e., illusory recollection). In this context, it is difficult to control for the veracity of the personal memories recalled by NDErs. Nonetheless, we believe that the production of false memories and illusory recollections would have affected the three subtypes of memories equally. Indeed, some factors such as age and affect were shown to impact the emergence of false memories. Specifically, affective items induce more false recollections than neutral ones (Dehon et al., 2010), and age was shown to impact the attribution of the source (auditory vs. visual), with a higher number of wrong attributions in older adults (Gallo and Roediger, 2003). Given the design of our study (i.e., within subject measures) and the temporal proximity between events, the three types of memories should have been impacted similarly by the age of participants. Regarding affect, all memories were highly emotional, albeit autobiographical and NDE memories were associated with positive emotions and flashbulb memories with negative ones. Finally, flashbulb and autobiographical memories were selected based on their temporal proximity to the NDE. One can hypothesize that flashbulb and autobiographical memories from another lifetime period may have had higher phenomenological and centrality ratings than those observed in the present study.

## Conclusion

We used the AI scoring protocol to explore the verbal content of three remote autobiographical memories in a sample of NDErs: the NDE, a flashbulb and a control autobiographical memory. Overall, NDE verbal recollections include a higher overall amount of details and a higher amount of internal/episodic details than flashbulb and autobiographical memories. More precisely, they comprise more event and perceptual internal details. Moreover, we found that flashbulb memories are associated to a lower intensity of feelings while remembering and a lower personal importance, and are less reactivated and less susceptible to be remembered from a first person perspective. Finally, NDE memories are more central than autobiographical memories, which in turn are more central than flashbulb memories. Besides, more central events contain more episodic verbal details. Overall, our results fall in line with existing literature that highlights the uniqueness of NDE memories.

## Data Availability Statement

The datasets generated for this study are available on request to the corresponding author.

## Ethics Statement

The studies involving human participants were reviewed and approved by Ethics Committee of the Faculty of Medicine of the University of Liège.

## Author Contributions

HC: conceptualization, methodology, formal analysis, investigation, data curation, writing ߝ original draft, writing ߝ review and editing, and visualization. EB: formal analysis, investigation, and writing ߝ review and editing. CB: conceptualization, methodology, and supervision writing ߝ review and editing. NP: formal analysis and writing ߝ review and editing. VC-V: conceptualization and writing ߝ review and editing. SL: supervision, writing ߝ review and editing, and funding acquisition. CM: conceptualization, methodology, writing ߝ review and editing, supervision, and funding acquisition.

## Conflict of Interest

The authors declare that the research was conducted in the absence of any commercial or financial relationships that could be construed as a potential conflict of interest.
